# Single skyrmion true random number generator using local dynamics and interaction between skyrmions

**DOI:** 10.1038/s41467-022-28334-4

**Published:** 2022-02-07

**Authors:** Kang Wang, Yiou Zhang, Vineetha Bheemarasetty, Shiyu Zhou, See-Chen Ying, Gang Xiao

**Affiliations:** Department of Physics, Brown University, Providence, RI 02912 USA

**Keywords:** Spintronics, Magnetic devices

## Abstract

Magnetic skyrmions are of great interest to both fundamental research and applications in post-von-Neumann computing devices. The successful implementation of skyrmionic devices requires functionalities of skyrmions with effective controls. Here we show that the local dynamics of skyrmions, in contrast to the global dynamics of a skyrmion as a whole, can be introduced to provide effective functionalities for versatile computing. A single skyrmion interacting with local pinning centres under thermal effects can fluctuate in time and switch between a small-skyrmion and a large-skyrmion state, thereby serving as a robust true random number generator for probabilistic computing. Moreover, neighbouring skyrmions exhibit an anti-correlated coupling in their fluctuation dynamics. Both the switching probability and the dynamic coupling strength can be tuned by modifying the applied magnetic field and spin current. Our results could lead to progress in developing magnetic skyrmionic devices with high tunability and efficient controls.

## Abstract (web summary)

Skyrmions, a kind of topological spin texture, have been considered as having potential for use in spin-based computing. Here, Wang et al. study the local dynamics of skyrmions interacting with pinning sites, demonstrating a skyrmion true random number generator with the magnetic field and spin current as two control parameters.

## Introduction

In-memory and unconventional computing architectures receive considerable interest for their relevance in solving problems that von Neumann computers fail to address efficiently. Spintronic systems are attractive for hardware implementations of low-energy-consumption and high-speed computing architectures^1–5^. Spintronic devices such as stochastic magnetic tunnel junctions (MTJs)^1–3^ and nano-oscillators^4,5^ have been proven effective in addressing the issues of optimization, invertible logic and recognition. It is challenging, however, to integrate computing with data storage in a single die and achieve efficient communication, owing to different multilayer structures or growing conditions for the separate parts.

Magnetic skyrmions are topologically protected quasi-particles^6–12^ of great interest to both fundamental research and technological applications. Of particular interest is their potential for applications in In-memory and unconventional computing devices^13–20^. Magnetic skyrmions exhibit many desirable properties including stability, small size and highly efficient controllability, which make them effective carriers in racetrack memory devices^21,22^. More recently, micromagnetic simulations have shown that the dynamic motion of skyrmions, either in the presence of spin currents or thermal effects, offers a valuable opportunity to construct skyrmionic logic^23,24^ and unconventional computing^14–17,20^ architectures. Little experimental progress, however, has been reported in the implementation of skyrmionic devices^18,19^ owing to challenges in the precise control over skyrmion motion. Moreover, devices based on skyrmion motion usually exhibit geometric and operational complexities. Research into the functionalities of skyrmions in addition to their dynamic motion is needed to successfully implement skyrmionic devices.

**Fig. 1:**
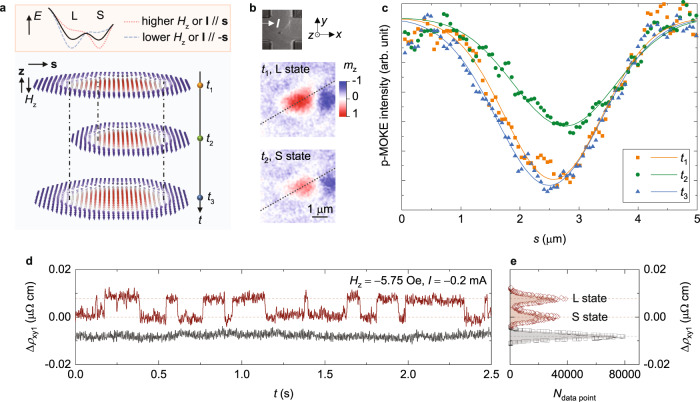
Local dynamics of a magnetic skyrmion. **a** Schematic of fluctuations of an isolated skyrmion interacting with local pinning centres in time between a small-skyrmion (S, at time *t*_2_) and a large-skyrmion (L, at times *t*_1_ and *t*_3_) state. Blue arrows correspond to downward orientations of magnetization (−*z* axis) whereas red arrows represent upward orientations of magnetization (*z* axis). The top panel shows a schematic of the energy landscape between two skyrmion states separated by an energy barrier which can be tuned by modifying the applied magnetic field *H*_*z*_ and the electric current *I*. **b** Polar-magneto optic Kerr effect microscope (p-MOKE) images of the two states of an isolated skyrmion. **c** P-MOKE signals along dotted lines in **b** (*s* increases from the left to right). Solid curves are fits to Gaussian functions. **d** Relative Hall-resistivity (∆*ρ*_xy1_) variations in time for a single skyrmion (upper) and a ferromagnetic state (bottom). **e** Statistics of data point distributions (*N*_data point_), open dots) as a function of ∆*ρ*_xy1_. Open dots are fitted to Gaussian functions. All results are measured at 307.1 K and a current *I* = −0.2 mA. Results for the skyrmion in **b**–**d** are measured at *H*_*z*_ = −5.75 Oe and for the ferromagnetic state at *H*_*z*_ = −5.89 Oe.

In this work we show that the local dynamics of skyrmions, in contrast to the global dynamics of a skyrmion, can overcome the above limitations and provide effective functionalities for versatile computing. The local dynamics of skyrmions can be introduced by local pinning centres which also enable the reliable positioning of skyrmions. These pinning centres can arise naturally in the sample growth process via intrinsic irregularities such as surface roughness, grain structures, and material composition variations with local variations in magnetic properties^25–27^. Additional artificial pinning centres can also be implemented experimentally by varying the film thickness in different regions^28^. One possible case of a skyrmion interacting with local pinning centres is schematically presented in Fig. 1a in which a pinning centre strongly pins one part of the skyrmion while the other part, under thermal effects, can fluctuate in time between two weaker pinning sites. These two states that we refer to as the small-skyrmion (S) and large-skyrmion (L) states occur at local minima in the energy landscape separated by an energy barrier (top panel in Fig. 1a). The energy landscape can be tuned by modifying the competing energies which determine the switching probability and fluctuation rate. This is similar to the behaviour of thermally-excited hopping of domain walls between pinning sites^29–31^ and the binary stochastic neurons as modelled by stochastic MTJs^3,32^. Intriguing features of skyrmions include their mobility in magnetic films and their mutual interactions which may provide additional functionalities.

In this work, we study the local dynamics of a single skyrmion and neighbouring skyrmions in a magnetic multilayer of substrate/Ta(1.6 nm)/Co_40_Fe_40_B_20_(CoFeB)(0.95 nm)/MgO(1.6 nm)/TaO_x_(2.0 nm) grown using magnetron sputtering (‘Methods’, see Supplementary Note I and Supplementary Fig. 2 for magnetic properties of the magnetic multilayer). This magnetic multilayer hosts skyrmions (see Supplementary Note II and Supplementary Fig. 3 for evidence of skyrmions) with a large size (diameter 1.0–2.2 µm, Supplementary Note III and Supplementary Fig. 5) compared to the size of pinning centres (on the order of 10 nm, Supplementary Note III^26^). We confirm that these magnetic bubbles are topologically stabilized skyrmions through observing the skyrmion Hall effect by which the skyrmions experience an additional transverse motion in addition to the longitudinal motion along the current-induced driving force direction (Supplementary Note II and Supplementary Fig. 3)^22,33^. Moreover, the skyrmions exhibit a left-handed Néel component of domain walls, as indicated by the asymmetric expansion of domain walls along the direction of an in-plane magnetic field (Supplementary Note II and Supplementary Fig. 4)^34–36^.

To implement moderate pinning strengths we carefully control the sputter rate of the Ta layer by regulating the DC power for deposition, as this is critical for the uniformity of magnetic parameters including the interfacial perpendicular magnetic anisotropy (PMA)^35,37^ and the Dzyaloshinskii-Moriya interaction (DMI)^35,38,39^. A slower sputter rate induces strong pinning of magnetic skyrmions whereas skyrmions in magnetic films grown with a higher sputter rate can move freely under thermal effects (Supplementary Note IV and Supplementary movies 1–3). The pinning effect arising from the local lattice distortion field owing to the spin-lattice coupling^40^ should be negligible as the magnetic film possesses a polycrystalline or amorphous structure. Due to the inherent spatial non-uniformities of pinning strengths, magnetic films with moderate growth conditions host skyrmions in which one part of the skyrmion is pinned strongly while the rest of it fluctuates in time between two weaker pinning centres (Fig. 1b–d, see Supplementary Note V and Supplementary Fig. 6 for more examples of local dynamics of skyrmions), as shown in Fig. 1a. We study the local dynamics of skyrmions both through direct imaging using a polar-magneto optic Kerr effect (p-MOKE) microscope and through Hall-resistivity measurements using a Hall cross with dimensions 20 × 20 μm^2^ (‘Methods’ and Supplementary Fig. 1). The Hall resistivity *ρ*_xy_ is dominated by the anomalous Hall resistivity which is proportional to the perpendicular magnetization while the ordinary and topological Hall resistivities are negligible in comparison.

## Local dynamics of a single skyrmion

We first explore the local dynamics of an isolated skyrmion that we intentionally create in the Hall cross (‘Methods’, Fig. 1b–d). Figure 1b–d illustrates the typical p-MOKE (Supplementary movie 4) and electronic measurement results of a skyrmion when applying a perpendicular magnetic field *H*_*z*_ = −5.75 Oe and an electric current *I* = −0.2 mA. The positive values refer to the field and current vectors along the *z* and *x* axes, respectively, and the negative values refer to those along the –*z* and –*x* axes, respectively (Fig. 1b). The p-MOKE images (Fig. 1b) along with the line sections of p-MOKE signals (Fig. 1c) indicate that the contraction of the skyrmion from the L to S state or expansion from the S to L state is dominated by the motion of the left part of the skyrmion while the right part of the skyrmion is more strongly pinned. This variation in skyrmion size results in the fluctuation of the Hall-resistivity signals as shown in Fig. 1d. The relative Hall resistivities of the saturated ferromagnetic state (∆*ρ*_xy1_ ≈ −0.008 μΩ cm) and the S (∆*ρ*_xy1_ ≈ 0) and L (∆*ρ*_xy1_ ≈ 0.008 μΩ cm) states of the skyrmion are consistent with the variations in the perpendicular magnetization between the three states as observed in our p-MOKE measurements. Note that although the Hall-cross area is much larger than the skyrmion size (Fig. 1b), electronic signals of the two states of an isolated skyrmion are distinguishable (Fig. 1d). The electronic signals can be enhanced by reducing the active area relative to the skyrmion size. We also note that the fluctuation in skyrmion size follows a purely random process as inferred from the histogram of the switching event time (Supplementary Note VI and Supplementary Fig. 7). This suggests that an isolated skyrmion is a good candidate for the true random number generator (TRNG) with the perpendicular magnetic field *H*_*z*_ and the current *I* serving as effective controls for the switching (studied below). To quantify the switching, we plot the statistics of data point distributions in Hall-resistivity measurements as a function of ∆*ρ*_xy1_ (Fig. 1e). Each peak corresponding to the S or L state can be fitted to a Gaussian function, and the areas under these curves are used to calculate the switching probability *p*_S_ at the S state and *p*_L_ at the L state.

We then study how the switching of the skyrmion can be tuned by modifying the competing energies. On the one hand, the perpendicular magnetic field *H*_*z*_ controls the energy landscape through the Zeeman energy such that a lower field tends to stabilize the L-state skyrmion while a higher field stabilizes the S-state skyrmion. On the other hand, the spin current^41,42^ ${{{{{{\boldsymbol{J}}}}}}}_{{{{{{\rm{s}}}}}}}=\frac{\hslash }{2e}{\Theta }_{{{{{{\rm{SH}}}}}}}\left({{{{{\boldsymbol{\sigma }}}}}}\times {{{{{{\boldsymbol{J}}}}}}}_{{{{{{\rm{e}}}}}}}\right)$ which can be derived from the electric current ***J***_*e*_ flowing through the spin Hall material, Ta, is also an effective tool to control the magnetization. Here, ℏ is the reduced Planck constant, *e* is the elementary charge, ${\Theta }_{{{{{{\rm{SH}}}}}}}$ is the spin Hall angle of the Ta layer, and ***σ*** is the spin polarization unit vector of the spin current. The spin current exerts spin torques including the field-like torque ***τ***_FL_ = *ζ*_FL_***m*** × ***σ*** and damping-like torque ***τ***_DL_ = *ζ*_DL_***m*** × (***m*** × ***σ***) on the magnetization where *ζ*_FL_ and *ζ*_DL_ are the field-like and damping-like torque coefficients, respectively^41,42^, and ***m*** is the normalized magnetization. The electric current flowing through the CoFeB layer can also be spin-polarized by the magnetization and exerts a Zhang-Li spin-transfer torque on the magnetization^43^. This spin-transfer torque, however, has been demonstrated to play a negligible role in the domain-wall and skyrmion motions in comparison to the spin-orbit torques ***τ***_FL_ and ***τ***_DL_, as demonstrated in our previous work^35^. The mobile part of the skyrmion, under the influence of these spin torques, tends to move along the direction of the electric current with an additional transverse motion owing to the Magnus force induced by the topology^35,44,45^. More specifically, the motion of the mobile part of the skyrmion is determined mainly by the pinning sites and may deviate from the intrinsic skyrmion Hall angle.

**Fig. 2:**
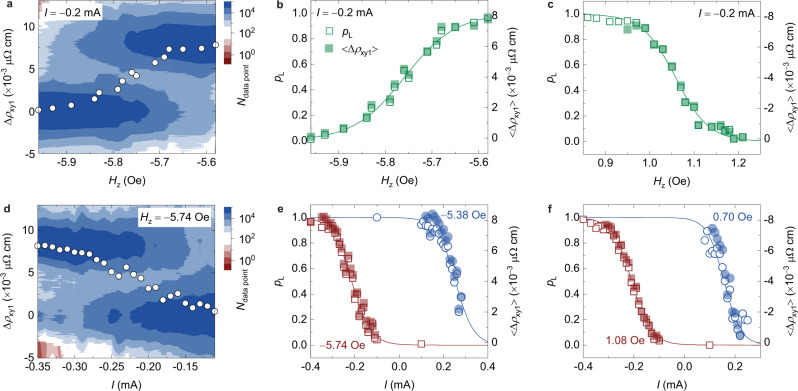
Field and current control of the switching. **a** Statistics of data point distributions as a function of ∆*ρ*_xy1_ in the presence of different fields *H*_*z*_ and at a constant current *I* = −0.2 mA. Open dots represent the results of time-averaged ∆*ρ*_xy1_, 〈∆*ρ*_xy1_〉. **b**, **c** The field *H*_*z*_ dependencies of the switching probability *p*_L_ (open dots) and 〈∆*ρ*_xy1_〉 (solid dots). **d** Statistics of data point distributions with different currents *I* and at a constant field *H*_*z*_ = −5.74 Oe. **e**, **f** Current *I* dependencies of *p*_L_ (open dots) and 〈∆*ρ*_xy1_〉 (solid dots) at different fields. Lines are sigmoidal fits. All results are measured at 307.1 K. Results in **a**, **b**, **d**, **e** are for the skyrmion shown in Fig. 1b with upward orientations of magnetization (*z* axis) in the skyrmion core. Results in **c** and **f** are for the skyrmion with a reversed polarity (downward orientations of magnetization (−*z* axis) in the skyrmion core) created at the same position.

Figure 2a–c shows the field control of this switching when applying an electric current *I* = −0.2 mA while Fig. 2d–f illustrates the switching dependence on the current. Figure 2a summarizes the statistics of data point distributions as a function of ∆*ρ*_xy1_ in the presence of different field strengths. The peaks at ∆*ρ*_xy1_ ≈ 0.008 μΩ cm (L state) and at ∆*ρ*_xy1_ ≈ 0 (S state) attenuate and amplify, respectively, when the field is swept from −5.58 to −5.96 Oe. This corresponds to the transition of the skyrmion from the L state (*p*_L_ ≈ 1) to the S state (*p*_L_ ≈ 0) through stochastic behaviour at the middle field, as illustrated in Fig. 2b where the time-averaged ∆*ρ*_xy1_, 〈∆*ρ*_xy1_〉 is also displayed. The field control of this switching has also been observed for a skyrmion with a reversed polarity created at the same position (Fig. 2c). The field control of this switching is a consequence of the Zeeman-energy variation and can be well fitted to the sigmoidal function. At a fixed field, a varying electric current *I* can also induce transitions of the skyrmions between the S and L states as illustrated in Fig. 2d–f. This transition between the two states occurs as the field is changed by an amount as small as 0.3 Oe (Fig. 2a–c) or as the current is changed by an amount as small as 0.25 mA (Fig. 2d–f). This suggests that the perpendicular field *H*_*z*_ and the current *I* are two highly sensitive control parameters for the switching of a skyrmion which can serve as a robust TRNG. To the best of our knowledge, this is the first experimental implementation of the skyrmion-based TRNG which is in great demand for probabilistic computing. In response to a recent experimental report of a reshuffle device based on the Brownian motion of multiple skyrmions^18^, a TRNG based on the local dynamics of a single skyrmion is more spatially compact and eliminates the need for a reshuffle device in probabilistic computing. Furthermore, unlike other stochastic neurons as modelled by stochastic MTJs^3,32^, magnetic skyrmions possess novel characteristics including the ability to move between sites, reverse polarities and interact with other neighbouring skyrmions (studied below) in addition to a high sensitivity of the switching probability to the applied field and current.

## Dynamic coupling between neighbouring skyrmions

**Fig. 3:**
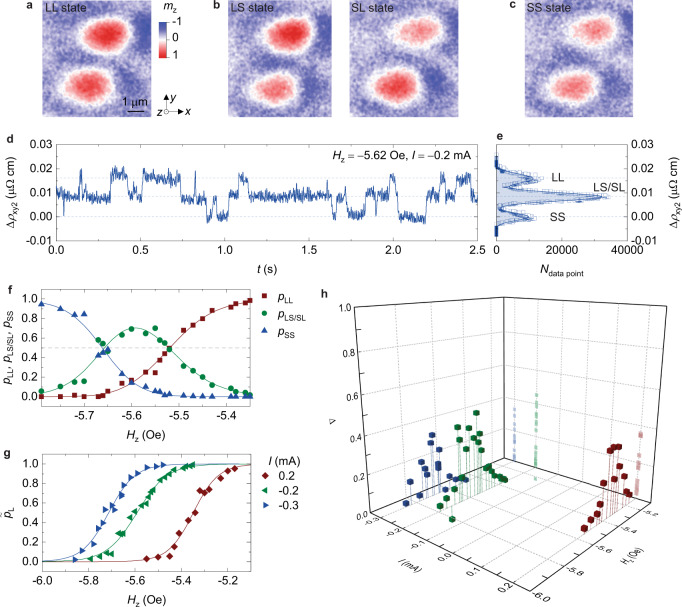
Dynamic coupling between neighbouring skyrmions. **a**–**c** P-MOKE images of two neighbouring skyrmions at the LL, LS/SL and SS states, respectively. **d** Relative Hall-resistivity (∆*ρ*_xy2_) variations in time for the two skyrmions in (**a**–**c**). **e** Statistics of data point distributions (*N*_data point_), open dots) as a function of ∆*ρ*_xy2_. Open dots are fitted to Gaussian functions. The measurement is performed at *H*_*z*_ = −5.62 Oe and *I* = −0.2 mA. **f** Field dependencies of *p*_*SS*_, *p*_LS/SL_ and *p*_LL_ probabilities at a current *I* = −0.2 mA. Lines are to guide the eye. **g**, **h** The field and current dependencies of ${\widetilde{p}}_{{{{{{\rm{L}}}}}}}$ (**g**) and ∆ (**h**) derived from *p*_SS_, *p*_LS/SL_ and *p*_LL_ probabilities. Lines in **g** are sigmoidal fits. All results are measured at 307.1 K.

To study how the local dynamics of neighbouring skyrmions interact, we create two skyrmions separated by about 3 μm from core to core as a model of the simplest skyrmion network (Fig. 3a–c). The two skyrmions under study, independently, exhibit similar size variation (Supplementary Fig. 8) as well as field and current dependencies of the switching (Supplementary Fig. 9). Interacting with local pinning centres at a moderate field *H*_*z*_ and current *I*, the skyrmion pair can fluctuate in time between four discrete states: LL (Fig. 3a), LS (Fig. 3b, left panel), SL (Fig. 3b, right panel) and SS (Fig. 3c) states, as supported by both p-MOKE (Supplementary movie 5) and Hall-resistivity (Fig. 3d) measurements. In this notation, the first L or S represents the state of the upper skyrmion in Fig. 3a–c, while the second L or S represents the state of the bottom skyrmion. As in the study of an isolated skyrmion, we extract the probabilities *p*_SS_ of being at the SS state, *p*_LS/SL_ at the LS/SL states and *p*_LL_ at the LL state via the statistics of data point distributions in the Hall-resistivity measurements (Fig. 3e). Figure 3f shows the *p*_SS_, *p*_LS/SL_ and *p*_LL_ probabilities at different fields *H*_*z*_ and a constant current *I* = −0.2 mA. We notice that at the middle field *p*_LS/SL_ ≈ 0.70 and *p*_LL_ ≈ *p*_SS_ ≈ 0.15. This could be evidence that the two skyrmions are linked by an anti-correlated coupling in which one skyrmion has an increased probability at the L or S state while the other skyrmion is at the S or L state. Additionally, through p-MOKE measurements, we observe that the two skyrmions spend most of the time fluctuating between the LS and SL states (Supplementary movie 5 and Supplementary Fig. 10). Measuring the local dynamics of the two skyrmions using different currents (0.2 and −0.3 mA) yields similar results (Supplementary Fig. 11). This anti-correlated coupling is most likely mediated by the demagnetization field while the coupling^46^ induced by the overlap between spin textures of the two skyrmions is negligible due to their large separation. More specifically, when one skyrmion is at the S or L state, the demagnetization field it generates at the other skyrmion position along the normal direction antiparallel to the magnetization direction at the skyrmion core would be smaller or larger, leading the other skyrmion to have an increased probability at the L or S state, respectively. We have calculated the demagnetization-field variation owing to the skyrmion size fluctuation (Supplementary Note VII and Supplementary Fig. 12). The results clearly show that when one skyrmion varies from the S to L state, or vice versa, the demagnetization field it generates at the other skyrmion position along the sample normal direction varies by an amount on the order of −0.3 or 0.3 Oe, respectively. This field variation is comparable to that required for the transition between *p*_L_ ≈ 0 and *p*_L_ ≈ 1 for an isolated skyrmion (Fig. 2a–c) and correspondingly leads to a higher probability of the other skyrmion to be in the S or L state.

We assume that the two skyrmions, independently, have the same field and current dependencies of the probability ${\widetilde{p}}_{{{{{{\rm{L}}}}}}}$ (${\widetilde{p}}_{{{{{{\rm{S}}}}}}}=1-{\widetilde{p}}_{{{{{{\rm{L}}}}}}}$) (Supplementary Fig. 9). Furthermore, we assume that when one skyrmion is at the L state, the other skyrmion has an increased probability of ${\widetilde{p}}_{{{{{{\rm{S}}}}}}}\left(1+\triangle \right)$ and a decreased probability of $1-{\widetilde{p}}_{{{{{{\rm{S}}}}}}}\left(1+\triangle \right)$ to be in the S and L states, respectively. On the other hand, when one skyrmion is at the S state, the other skyrmion has an increased probability of ${\widetilde{p}}_{{{{{{\rm{L}}}}}}}\left(1+\triangle \right)$ and a decreased probability of $1-{\widetilde{p}}_{{{{{{\rm{L}}}}}}}\left(1+\triangle \right)$ to be in the L and S states, respectively. The dimensionless parameter ∆ ranges from 0 to 1 and can be used to characterize the dynamic coupling strength between the two skyrmions. With the above assumptions, ${\widetilde{p}}_{{{{{{\rm{L}}}}}}}$ and ∆ can be extracted from$\,{p}_{{{{{{\rm{SS}}}}}}}={\widetilde{p}}_{{{{{{\rm{S}}}}}}}-{\widetilde{p}}_{{{{{{\rm{L}}}}}}}{\widetilde{p}}_{{{{{{\rm{s}}}}}}}\left(1+\triangle \right)$, ${p}_{{{{{{\rm{LS}}}}}}/{{{{{\rm{SL}}}}}}}=2{\widetilde{p}}_{{{{{{\rm{L}}}}}}}{\widetilde{p}}_{{{{{{\rm{s}}}}}}}\left(1+\triangle \right)$ and ${p}_{{{{{{\rm{LL}}}}}}}={\widetilde{p}}_{{{{{{\rm{L}}}}}}}-{\widetilde{p}}_{{{{{{\rm{L}}}}}}}{\widetilde{p}}_{{{{{{\rm{s}}}}}}}\left(1+\triangle \right)$ and are illustrated in Fig. 3g, h. For the two skyrmions under study, at a fixed current, the coupling strength peaks at the middle field and weakens by either increasing or decreasing the field (Fig. 3h). Because the demagnetization-field variation that results from the fluctuation in skyrmion size is independent of the field and current, a decrease in the ∆ value can be explained by a decrease in the switching-probability sensitivity to the field variation (Fig. 2b, c). Contrastingly, both the field dependencies of ${\widetilde{p}}_{{{{{{\rm{L}}}}}}}$ and ∆ shift to a higher field when decreasing the current, due to the combined effects of the Zeeman-energy variation and spin-orbit torque.

The considerable dynamic coupling between neighbouring skyrmions, which has not been observed in other stochastic neurons, results from the high sensitivity of the switching probability of a single skyrmion to the magnetic field *H*_*z*_ (Fig. 2a–c). Although a recent experimental work reports dynamic coupling between two stochastic MTJs using an electrical circuit^47^, the coupling strength is low and a more complex electrical circuit design is required to dynamically couple multiple MTJs. Contrastingly, substantial dynamic coupling may exist in a complex skyrmion network with multiple skyrmions. The dynamic coupling between neighbouring skyrmions makes skyrmion networks strong candidates for applications in logic operations, reservoir computing and Ising computing devices (Supplementary Note VIII and Supplementary Fig. 13). Efficient control over the switching probability and the dynamic coupling strength between neighbouring skyrmions by utilizing the magnetic field and current grants skyrmionic devices based on skyrmion networks high tunability.

## Discussion and outlook

**Fig. 4:**
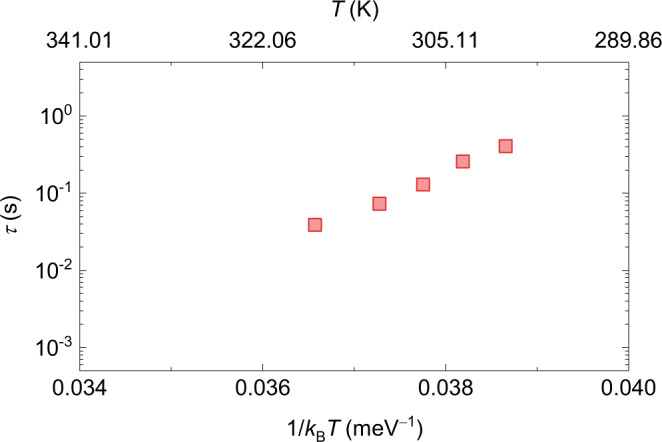
Average residence times in the local dynamics of a skyrmion. The average residence time *τ* of a skyrmion pinned in a L or S state shown in Fig. 1b as a function of the temperature *T*.

The fluctuation rate *f* between the two states of a skyrmion determines the operation speed of skyrmionic devices. Figure 4 plots the average residence time *τ* (*τ* = 1/*f*) of the skyrmion shown in Fig. 1b as a function of temperature *T*. It can be concluded that the average residence time decreases with increasing temperature. In addition to bringing about thermal activation, the temperature also affects magnetic properties of the magnetic thin film. Parameters such as saturation magnetization *M*_S_, exchange stiffness *A*, DMI *D*_Int_, and PMA *K*_u_ decrease with increasing temperature as indicated by the scaling ${M}_{{{{{{\rm{S}}}}}}}={M}_{{{{{{\rm{S}}}}}}}(0)\left[1-{\left(T/{T}_{0}\right)}^{\alpha }\right]$, $A\propto {{M}_{{{{{{\rm{S}}}}}}}}^{\beta }$, ${D}_{{{{{{\rm{Int}}}}}}}\propto {{M}_{{{{{{\rm{S}}}}}}}}^{\gamma }$, and ${K}_{{{{{{\rm{u}}}}}}}\propto {{M}_{{{{{{\rm{S}}}}}}}}^{n(n+1)/2}$ where *α* = 3/2 and *β* = *γ* = 2 in a mean-field approximation, and *n* = 2 is the dimensionality of the magnetic film^48–52^. Correspondingly, the spatial variation in any one parameter is expected to be reduced at an elevated temperature, reducing the energy barrier ∆*E* between two skyrmion states. According to Arrhenius law *f* = *f*_0_exp(–Δ*E*/*k*_B_*T*), assuming a constant attempt frequency *f*_0_, both an increase in the thermal activation energy *k*_B_*T* and reduction in ∆*E* would cause a faster fluctuation rate. The attempt frequency, however, may carry an activation entropy^53–56^ and can be expressed by *f*_0_ = *f*_00_exp(Δ*E*/*E*_0_ + *b*) if the Meyer-Neldel rule applies^57–59^. Here, *E*_0_ is a characteristic energy, *f*_00_ is a positive prefactor, and *b* is a constant. In this scenario, the fluctuation rate may have a weaker energy-barrier dependency.

In addition to increasing the thermal activation energy and reducing the energy barrier between two skyrmion states, reducing the distance ∆*q* between two weaker pinning sites may also increase the fluctuation rate. This is a result of the entropic effect which implies that a longer path must be explored more randomly while the energy barrier may remain the same (see Supplementary Note IX for more discussions^54–56^). We note that from micromagnetic simulations in which we introduce grains with an average size of 10 nm and random distributions of the PMA and DMI, we observe an average residence time as low as 30 ns for the mobile part of the skyrmion fluctuating in time between two pinning sites that are separated by ~50 nm (‘Methods’, Supplementary Note X and Supplementary Fig. 14). We infer from this that a fluctuation rate beyond the MHz range can be experimentally achieved by more elaborate control of the energy landscape such as through the implementation of artificial pinning centres which can arise from thickness modulations, voids in multilayers, embedded impurity atoms or adatoms adhering to the surface introduced with advanced thin-film fabrication, lithography, irradiation, ion implantation or laser ablation techniques^60^.

In contrast with previous efforts to understand global dynamics of magnetic skyrmions, we have studied for the first time their local dynamics which demonstrate effective functionalities for versatile computing. In addition to the local dynamics of a single skyrmion which allows for the unprecedented implementation of a skyrmion-based TRNG for probabilistic computing, we also demonstrate an anti-correlated coupling between neighbouring skyrmions. Both the local dynamics and the dynamic coupling strength can be effectively controlled by modifying the applied magnetic field and current. This attribute offers opportunities to implement skyrmionic devices based on either a single skyrmion or a skyrmion network with a higher degree of tunability and controllability. We note that the local dynamics of skyrmions studied here refer to one specific scenario. Other local dynamics such as the fluctuation of a skyrmion between three discrete states may also be introduced by manipulating the spatial variations in the energy landscape. Furthermore, the local dynamics of skyrmions are expected to be observed in various magnetic systems such as ferrimagnets where magnon serves as a more energy-efficient control. With a better understanding of the local dynamics of skyrmions, versatile skyrmionic devices can be designed and realized experimentally.

## Methods

### Sample preparation

We deposit multilayers of Ta(1.6 nm)/Co_40_Fe_40_B_20_(CoFeB)(0.95 nm)/MgO(1.6 nm)/TaO_x_(2.0 nm) on thermally oxidized silicon wafers using a high-vacuum magnetron sputtering system with a base vacuum pressure of 5.0 × 10^−8^ Torr. The MgO layer is deposited using radio-frequency (RF) power with an argon pressure of 0.7 mTorr while other layers are deposited using DC power with an argon pressure of 1.4 mTorr. We use 15 W of DC power for the deposition of the ferromagnetic CoFeB layer and a lower DC power (3, 4 and 5 W) for the deposition of the Ta layers. The DC power for the Ta-layer deposition is regulated to implement moderate pinning of magnetic skyrmions (Supplementary Note IV). We pattern the samples into Hall crosses with dimensions of 20 × 20 μm^2^ using photolithography and physical ion milling. The samples are then annealed at different temperatures in a high-vacuum chamber with a vacuum pressure of ~1.0 × 10^−6^ Torr in the presence of a magnetic field of ~0.4 T normal to the sample plane.

### Experimental methods

A schematic of the experimental setup is presented in Supplementary Fig. 1. The sample is placed on a printed circuit board (PCB) and is connected to the PCB using a wire-bonder machine (HB10, TPT), which is further connected to external circuits as shown in Supplementary Fig. 1b. A polyimide flexible heater is placed on the bottom of the PCB to heat the sample while a thermal via is introduced in the PCB to enhance the thermal conductivity between the heater and the sample. An electromagnet placed under the PCB is used to supply a perpendicular magnetic field. The temperature and the magnetic field are detected via a temperature sensor and a Hall sensor, respectively, which are both placed close to the sample on the PCB. We wait for a sufficiently long time before collecting measurements to ensure the sample temperature is stable.

We study magnetic skyrmions through both direct imaging and electronic methods. A home-made polar-magneto optic Kerr effect (p-MOKE) microscope is used to record magnetic images. This p-MOKE setup consists of a 633 nm wavelength (*λ*) laser and a 50× objective lens with a numerical aperture (*NA*) of 0.80. Consequently, the maximum achievable resolution is about 0.61*λ*/*NA* = 0.48 μm which is notably smaller than the size of skyrmions in this study (see Supplementary Note III and Supplementary Fig. 5). In p-MOKE measurements, we try to achieve the best resolution of the microscope. The skyrmion profile as shown in Fig. 1c is extracted directly from the map recorded by the CCD camera. The average skyrmion diameter is derived from measurements on multiple skyrmions in p-MOKE images with the known length scale of the Hall cross as a reference.

Additionally, we detect electronic signals by measuring Hall voltages which are intensified through amplifiers (INA828, Texas Instruments). The amplified signals from two independent amplifiers are converted to digital signals through a high-speed data acquisition (DAQ) device (USB-1602HS, Measurement Computing) with 16-bit resolution and a high sample rate of 2 MS/s. A digital low-pass filter at 100 Hz is applied to the averaged signals from the two channels to minimize noise. This electronic method allows us to detect electronic signals from a single skyrmion.

### Skyrmion creation

Magnetic skyrmions can be transformed from a labyrinthine domain phase by either increasing or decreasing the field (Supplementary Fig. 2). In addition to the field, a spin current can also lead to the transformation from a labyrinthine domain phase to the state with multiple skyrmions^35^. In this study, multiple skyrmions are created by the combined effects of the magnetic field and the spin current. Skyrmions are nucleated at pinning centres^12^ and their distributions may differ from the application of one current pulse to another. Due to spatial non-uniformities of the pinning strengths, the critical current or the field required for escape from the pinning centres varies for different skyrmions. To focus on a particular skyrmion, we first nucleate multiple skyrmions in which the targeted skyrmion exists and intentionally remove other skyrmions by applying different currents or by varying the external field.

### Micromagnetic simulations

We perform micromagnetic simulations using finite-difference solver MUMAX3 based on the graphic processing unit^61^. The time-dependent normalized magnetization ***m*** is obtained from solving the Landau–Lifshitz–Gilbert (LLG) equation. In micromagnetic simulations, the magnetic parameters we adopt include the saturation magnetization *M*_S_ = 1.06 × 10^6^ A/m, the exchange stiffness *A* = 1.5 × 10^−11^ J/m, the averaged PMA *K*_u_ = 7.58 × 10^5^ J/m^3^, the averaged DMI constant *D*_Int_ = 0.193 × 10^−3^ J/m^2^, and the Gilbert damping constant *α* = 0.1. The magnetic parameters that we chose to use in the current micromagnetic simulations are determined by our previous measurements and describe the magnetic materials used in this study well due to the similar multilayer structure and growth conditions^35^. To implement pinning centres, we introduce grains with an average size of 10 nm and random distributions of the interfacial PMA and DMI. We set the random PMA variation at $\frac{\triangle {K}_{{{{{{\rm{u}}}}}}}}{{K}_{{{{{{\rm{u}}}}}}}}=7 \%$ and the random DMI variation at$\,\frac{\triangle {D}_{{{{{{\rm{Int}}}}}}}}{{D}_{{{{{{\rm{Int}}}}}}}}=21 \%$. We also account for thermal fluctuations at 300 K by adding a stochastic thermal field into the LLG equation. The simulation results are presented in Supplementary Note X and Supplementary Fig. 14.

## Supplementary information

Supplementary Information

Supplementary movie 1

Supplementary movie 2

Supplementary movie 3

Supplementary movie 4

Supplementary movie 5

Description of Additional Supplementary Files

## Data Availability

The authors declare that the data supporting the findings of this study are available within the article and from the corresponding author upon reasonable request.
