# Supplementary material for: Single skyrmion true random number generator using local dynamics and interaction between skyrmions
Source: Nat Commun. 2022 Feb 7;13:722. doi: 10.1038/s41467-022-28334-4 (PMC8821635; doi:10.1038/s41467-022-28334-4)
Supplement: Supplementary file 1 — Supplementary Information [file 41467_2022_28334_MOESM1_ESM.pdf]

Supplementary Information for

Singles skyrmion true random number generator using local dynamics and interaction between skyrmions

Kang Wang*, Yiou Zhang, Vineetha Bheemarasetty, Shiyu Zhou, See-Chen Ying, Gang Xiao*

* Email: kang_wang@brown.edu; gang_xiao@brown.edu

Contents

I. Magnetic properties of magnetic films

II. The evidence of magnetic skyrmions – skyrmion Hall effect

III. The skyrmion size and pinning size

IV. Effects of the sputter rate on the pinning of magnetic skyrmions

V. More examples of local dynamics of skyrmions

VI. Histogram of the switching event time

VII. Demagnetization-field variation owing to the fluctuation in skyrmion size

VIII. Potential of local dynamics of skyrmions for applications in computing

IX. Fluctuation rate in the local dynamics of a skyrmion

X. Micromagnetic simulations of local dynamics of skyrmions

I. Magnetic properties of magnetic films

Magnetic configurations in magnetic multilayers are determined by the competition between the Zeeman (U_{Zeeman}), exchange (U_{ex}), anisotropy (U_{ani}), Dzyaloshinskii-Moriya interaction (DMI) (U_{DMI}) and demagnetization (U_{demag}) energies

$$
\begin{aligned}
U &= U_{\mathrm{Zeeman}} + U_{\mathrm{ex}} + U_{\mathrm{ani}} + U_{\mathrm{DMI}} + U_{\mathrm{demag}} \\
&= -\mu_0 H_{\mathrm{z}} M_{\mathrm{S}} \int \mathrm{d}^2 x m_{\mathrm{z}} + A \int \mathrm{d}^2 x (\nabla \mathbf{m})^2 - K_{\mathrm{u}} \int \mathrm{d}^2 x m_{\mathrm{z}}^2 \\
&\quad + D_{\mathrm{Int}} \int \mathrm{d}^2 x [m_{\mathrm{z}} \mathrm{div}\, \mathbf{m} - (\mathbf{m} \cdot \boldsymbol{\nabla}) m_{\mathrm{z}}] - \frac{1}{2} \mu_0 M_{\mathrm{S}} \int \mathrm{d}^2 x \mathbf{m} \cdot \mathbf{H}_{\mathrm{demag}}.
\end{aligned}
\tag{S1}
$$

In Eq. S1, $\mathbf{m} = (m_{\mathrm{x}}, m_{\mathrm{y}}, m_{\mathrm{z}})$ is the normalized magnetization, H_{z} is the perpendicular magnetic field, M_{S} is the saturation magnetization, A is the exchange stiffness, K_{u} is the perpendicular magnetic anisotropy (PMA), D_{Int} is the DMI constant and $\mathbf{H}_{\mathrm{demag}}$ is the demagnetization field. Supplementary Figure 2 displays the magnetic-configuration dependence on the perpendicular magnetic field H_{z}, which is illustrated by both the Hall-resistance (R_{H}) measurement (Supplementary Fig. 2a) and p-MOKE images (Supplementary Fig. 2b). We observe a shift in the Hall curve from zero field which is the classic signature of exchange bias (Supplementary Fig. 2a). The exchange bias most likely arises from the CoFeB/MgO interface magnetization as discussed in detail in previous studies[1,2]. At the exchange bias field of -2.21 Oe, a labyrinthine multi-domain state is observed (Supplementary Fig. 2b). The labyrinthine domain phase transforms into the state with multiple skyrmions by either increasing or decreasing the field, which finally transforms into the uniform magnetization state at even higher or lower magnetic fields. The state with multiple skyrmions appears in the field range where an irreversible Hall curve is observed (Supplementary Fig. 2a).

II. The evidence of magnetic skyrmions – skyrmion Hall effect

Magnetic skyrmions are topologically protected quasi-particles with non-zero topological charges $Q = \pm 1$. Due to its topology, the skyrmion experiences a skyrmion Hall effect under a driving force. On biasing with a current, in addition to the longitudinal motion along the current direction, the skyrmion also acquires a transverse velocity component through a Magnus force induced by the topology[3,4]. The phenomenon of skyrmion Hall effect can be used to distinguish skyrmions from other trivial magnetic structures.

Here we consider the motion of a general magnetic texture. The centre of mass motion of the magnetic texture can be approximately described by the Thiele equation[5,6]

$$\mathcal{G} \times \boldsymbol{v} + \alpha\mathcal{D}\mathbf{v} + \frac{1}{2}\gamma_{\mathrm{L}}\beta\boldsymbol{\mathcal{T}} = 0. \tag{S2}$$

The first and second terms are functions of the velocity $\mathbf{v}$ of the magnetic texture and are known as Magnus force and dissipative force, respectively. The gyro coupling vector $\mathcal{G}$ only has a z-component $\mathcal{G} = 4\pi Q = 4\pi \int q(\mathbf{r})dxdy$ which is an integration of the topological charge density $q(\mathbf{r})$. The third term is the current-induced driving force for the motion of a magnetic texture, with the coefficient $\frac{1}{2}\gamma\beta = \frac{\gamma_{\mathrm{L}}|\Theta_{\mathrm{SH}}|\hbar}{2M_{\mathrm{S}}ed}$ and the vector $\boldsymbol{\mathcal{T}} = \int(\mathbf{e}_{\mathrm{z}} \times \mathbf{J}_{\mathrm{e}}) \cdot (\boldsymbol{\nabla}\mathbf{m} \times \mathbf{m})dxdy$, where γ_{L} is the gyromagnetic ratio, Θ_{SH} is the spin Hall angle of the spin Hall solid, $\hbar$ is the reduced Planck constant, e is the elementary charge, d is the ferromagnetic layer thickness, $\mathbf{e}_{\mathrm{z}}$ is the unit vector along the z axis, and $\mathbf{J}_{\mathrm{e}}$ is the charge current flowing through the spin Hall solid. For a rigid skyrmion, $\boldsymbol{\mathcal{T}} = \frac{\pi^2}{2}R\overleftrightarrow{\boldsymbol{\Gamma}}(\mathbf{e}_{\mathrm{z}} \times \mathbf{J}_{\mathrm{e}})$ with the tensor $\overleftrightarrow{\boldsymbol{\Gamma}} = \begin{bmatrix} 0 & -1 & 0 \\ 1 & 0 & 0 \\ 0 & 0 & 0 \end{bmatrix}$, and R is the skyrmion radius.

For magnetic skyrmions, the topological charge $Q = \pm 1$ where the sign depends on the polarity of the skyrmion. In this scenario, the Magnus force in Eq. S2 is non-zero, leading to a transverse motion of skyrmions accompanying the longitudinal motion along the current-induced driving force. The transversion motion direction reverses with reversing the polarity of skyrmions. Contrastingly, trivial magnetic bubbles with $Q = 0$ do not experience the Magnus force and thus the skyrmion Hall effect is invisible. Supplementary Figure 3a, b, illustrates the current-driven motion of skyrmions with both polarities which experience the skyrmion Hall effect. We also see that the skyrmion in Fig. 1b-d in the main text experiences the skyrmion Hall effect in the presence of a large current by which the skyrmion escapes from the local pinning centre, as shown in Supplementary Fig. 3c. The results confirm that the magnetic bubbles in this study are skyrmions with non-zero topological charges.

It is known that the chirality of magnetic domain walls depends on the relative values of the D_{Int} and $D_{\mathrm{thr}} = \frac{2\mu_0 M_{\mathrm{S}}^2}{\frac{\pi^2}{d\ln(2)} + \pi\sqrt{\frac{K_{\mathrm{u}}}{A}}}$.[7] The domain walls are Bloch type when $D_{\mathrm{Int}} = 0$. When $0 < |D_{\mathrm{Int}}| < D_{\mathrm{thr}}$, the domain walls belong to the mixed Bloch and Néel-types, and the domain walls become purely Néel-type when $|D_{\mathrm{Int}}| \geq D_{\mathrm{thr}}$. Previously, we have measured a magnetic thin film Ta(6.0 nm)/$Co_{40}Fe_{40}B_{20}$(1.0 nm)/ MgO(1.6 nm)/TaO_x(1.0 nm) hosting skyrmions[8]. We have demonstrated that $|D_{\mathrm{Int}}| \geq D_{\mathrm{thr}}$ and thus concluded purely Néel-type domain walls. We expect that the parameters of the magnetic thin film used for this study are close to our previous measurements as the multilayer structure and growth conditions are similar, and $|D_{\mathrm{Int}}| \geq D_{\mathrm{thr}}$ remains true.

To confirm the type of domain walls, we track evolutions of magnetic domains under an in-plane magnetic field, as presented in Supplementary Fig. 4. It has been shown that the Bloch-type domain walls would expand perpendicularly to the in-plane field while the expansion of the Néel-type domain walls is parallel to the in-plane field[9]. Domain walls with opposite vorticities also expand conversely. We first apply a perpendicular magnetic field, $H_z = -6.43$ Oe, to shrink the labyrinthine multi-domain phase into a mixed skyrmion and labyrinthine domain phase, and then apply an in-plane magnetic field H_{IP}. The asymmetric expansion of magnetic domains along the in-plane field direction suggests the left-handed Néel-type domain walls.

III. The skyrmion size and pinning size

Supplementary Figure 5 summarizes the skyrmion size at different temperatures and fields that are derived from p-MOKE images. The skyrmion size decreases with increasing field to lower the Zeeman energy. The skyrmion diameter D_{Skyr} in this study ranges from 1.0 to 2.2 μm and is similar to the size of skyrmions in Ta/CoFeB systems observed in previous studies[8,10].

We calculate the stable size of skyrmions D_{Skyr} ($D_{\mathrm{Skyr}} = 2R$) as a function of H_z through[11]

$$A\left(\frac{1}{w} - \frac{w}{R^2}\right) - \frac{\pi}{2} D_{\mathrm{Int}} + Kw + M_S H_z R = 0, \tag{S3}$$

$$A\left(-\frac{R}{w^2} + \frac{1}{R}\right) + KR + \frac{\pi^2}{12} M_S H_z w = 0. \tag{S4}$$

The skyrmion radius R and the domain-wall width w are two unknown parameters. Supplementary Fig. 5a-d display the calculation results with the adopted values of the effective PMA $K = K_u - \frac{1}{2}\mu_0 M_S^2$, M_S, A, and D_{Int}, as well as the experimental results. In calculations of each figure, only one parameter is varied while others are fixed. Supplementary Fig. 5 shows that the experimental results are roughly grouped on a series of lines calculated with adopting slightly variable parameters around $K = 11000\ \mathrm{J/m^3}$, $M_S = 1.06\ \mathrm{MA/m}$, $A = 15\ \mathrm{pJ/m}$, and $D_{\mathrm{Int}} = 0.193\ \mathrm{mJ/m^3}$. The M_S, A and D_{Int} parameters reflect our previous measurements[8]. The results suggest only small variations of the magnetic parameters within the narrow temperature range we study, as expected.

In the magnetic film under study, the pinning can be accounted for by the inherent inhomogeneities such as the surface roughness, grain structures, and/or material composition variations as a result of atomic diffusions at the interfaces. The inherent inhomogeneities may have a characteristic length comparable with

the grain size of the polycrystalline structure of the magnetic film. According to our x-ray diffraction measurements[12], the average grain size in the Ta layer is on the order of 10 nm which is much smaller than the skyrmion size. This suggests that a magnetic skyrmion can interact with a cluster of pinning centres, leading to the local dynamics as observed in this work.

IV. Effects of the sputter rate on the pinning of magnetic skyrmions

We regulate the DC power ($P_{\mathrm{Ta}} = 3, 4,$ and 5 Watt) for the deposition of the Ta layer to implement moderate pinning strengths. A lower DC power induces a lower sputter rate for the Ta layer. The Ta is known as a getter material. One expects that a lower sputter rate would induce more contaminations in the Ta layer. This could affect local atomic arrangements and increase spatial non-uniformities of the magnetic properties including the interfacial PMA and DMI. Indeed, we observe that the labyrinthine domains and skyrmions, under the influence of the current $I = 0.5$ mA, move negligibly in magnetic films grown with $P_{\mathrm{Ta}} = 3$ Watt, creep forward in magnetic films grown with $P_{\mathrm{Ta}} = 4$ Watt (Supplementary movie 1), and flow steadily in magnetic films grown with $P_{\mathrm{Ta}} = 5$ Watt (Supplementary movie 2). Moreover, we observe that skyrmions in magnetic films grown with a higher sputter rate ($P_{\mathrm{Ta}} = 5$ Watt) can move freely under thermal effects (Supplementary movie 3) which has not been observed in magnetic films grown with a lower sputter rate. The results confirm that a slower sputter rate causes stronger pinning of skyrmions. In this work, we study the local dynamics of skyrmions in a magnetic film grown with $P_{\mathrm{Ta}} = 4$ Watt corresponding to moderate pinning strengths.

V. More examples of local dynamics of skyrmions

Pinning centres and strengths are randomly distributed in naturally grown magnetic films. In the main text, we show the local dynamics of skyrmions interacting with a cluster including both strong and weak pinning centres. More examples of the local dynamics of skyrmions are illustrated in Supplementary Fig. 6. Local variations between the small-skyrmion (S) and large-skyrmion (L) states differ from one skyrmion to the other. Moreover, not all skyrmions in the magnetic film exhibit the local dynamics. There are also other scenarios. Some skyrmions are pinned by a cluster of strong pinning centres. In this scenario, no local dynamics have been observed and only a large current (> 0.6 mA) can drive these skyrmions to move away from the pinning centres. On the other hand, some skyrmions are pinned by a cluster of weak pinning centres and may escape from these pinning centres easily either under a small current or under thermal effects. Other local dynamics of skyrmions interacting with local pinning centres may also exist and remain to be further studied.

VI. Histogram of the switching event time

Fluctuations in skyrmion size between the S and L states are observed and studied via electronic measurements. The event time t_{event} is the time between subsequent switching events and varies between each event. We measure each event time from electronic measurements over 21 s. Supplementary Figure 7 displays an example of the histogram of the event time for the skyrmion shown in Fig. 1 in the main text, analysed at $H_{\mathrm{z}} = -5.75$ Oe and $I = -0.2$ mA. The number of events, N, shows an exponential decay with t_{event}, $N \propto e^{-t_{\mathrm{event}}/\tau}$. This indicates that the switching event follows a purely random process known as the Poisson process[13]. Exponential fittings to the histograms of switching event time for the S and L states of a skyrmion yield the average residence times τ_{S} and τ_{L}, respectively. The geometric average of these two residence times defined as $\tau \equiv \sqrt{\tau_{\mathrm{S}}\tau_{\mathrm{L}}}$ provides the average residence time τ shown in Fig. 4 in the main text.

VII. Demagnetization-field variation owing to the fluctuation in skyrmion size

The coupling between two neighbouring skyrmions is most likely mediated by the demagnetization-field variation that results from the fluctuation in skyrmion size. In this section, we calculate the demagnetization-field variation owing to the skyrmion-size variation. Supplementary Figure 12a, b, show the two neighbouring skyrmions at the LL and SS state, respectively, extracted from p-MOKE images. We then calculate the demagnetization field at other positions, generated by magnetic configurations in the green square region in Supplementary Figure 12a, b. In calculating the demagnetization field, we assume the magnetization is along the normal direction in all regions and that the perpendicular magnetizations inside and outside the skyrmion regions are $m_{\mathrm{z}} = 1$ and $m_{\mathrm{z}} = -1$, respectively. Moreover, we take the saturation magnetization $M_{\mathrm{S}} = 1.06 \times 10^{6}$ A/m into account. Supplementary Figure 12c displays the demagnetization-field variation $|\Delta H_{\mathrm{demag}}|$ owing to changes in magnetic configurations in green square regions in Supplementary Fig. 12a, b, at different positions along the red arrow. The results show that $|\Delta H_{\mathrm{demag}}|$ is on the order of magnitude of 0.3 Oe at the bottom-skyrmion position and is along the normal direction. The $|\Delta H_{\mathrm{demag}}|$ value is comparable to the field that is required for the switching-probability transition between $p_{\mathrm{L}} \approx 0$ and $p_{\mathrm{L}} \approx 1$ (Fig. 2 in the main text), indicating that the demagnetization-field variation due to the fluctuation in skyrmion size plays an important role in the anti-correlated coupling between the two skyrmions shown in Fig. 3 in the main text.

VIII. Potential of local dynamics of skyrmions for applications in computing

Supplementary Figure 13 schematically shows some potential applications of the skyrmion dynamics in computing. An isolated skyrmion can serve as a robust true random number generator for probabilistic computing (Supplementary Fig. 13a). Both the field and current are efficient parameters to control the switching probability of the probabilistic bit (Fig. 2 in the main text). The stochastic computer has been proven successful in addressing the issues of optimization and invertible logic that von Neumann computers fail to address efficiently[14]. Compared to other stochastic neurons as modelled by stochastic magnetic tunnel junctions (MTJs), intriguing features of skyrmions include their mobility in magnetic films and their mutual interactions which may provide additional functionalities. In construction of a probabilistic bit using a stochastic MTJ[14], a NMOS transistor was used to restrict the current applied to the system and a comparator was used to convert output signals to be either ideal 1 or 0. In construction of the probabilistic circuit, an Arduino microcontroller was used to read the output signals from comparators and implement the synaptic weights. Data acquisition (DAQ) was used to communicate with the microcontroller and convert the results into analogue voltages which were then sent back to the NMOS and the stochastic neuron. One expects that constructions of the probabilistic bit and circuit based on skyrmions can adopt a similar design.

If one can control each skyrmion using a local magnetic field or current, two neighbouring skyrmions with an anti-correlated coupling can serve as a strong candidate for logic devices (Supplementary Fig. 13b). Local magnetic fields can be applied through multiple methods such as through the stray field generated from a magnetized tip[15,16] or by applying an electric current to a strip patterned nearby the skyrmion locations[17]. The local current can be applied to individual skyrmions through patterning two Hall crosses with each Hall cross hosting one skyrmion. The magnetized tip can be integrated in the device like the read-write head in the hard disk drive. Additionally, advanced patterning techniques such as the e-beam lithography allow us to fabricate nanometre-sized structures such as the well-defined strip and Hall crosses. The local field or current can serve as inputs and the resultant Hall resistance serves as the output and can be detected through electronic measurements. In a logical AND operation, for example, inputs 1 and 0 can be currents for $p_{\mathrm{L}} = 1$ and $p_{\mathrm{L}} = 0.5$, respectively. When both inputs are 1, both skyrmions are at the L state, which gives a high resistivity of $\langle \Delta\rho_{\mathrm{xy2}} \rangle \approx 0.016$ μΩ cm corresponding to an output of 1. When the input for one skyrmion is 1 and the other is 0, one skyrmion would be stabilized at the L state while the other has an increased probability at the S state, leading to a lower resistivity of $\langle \Delta\rho_{\mathrm{xy2}} \rangle \approx 0.008$ μΩ cm which corresponds to an output of 0. When both inputs are 0, due to their mutual coupling, the two skyrmions are either at the LS or SL state, which also leads to a resistivity of $\langle \Delta\rho_{\mathrm{xy2}} \rangle \approx 0.008$ μΩ cm corresponding to an output of 0. In a logical OR operation, on the other hand, inputs 1 and 0 can be currents for $p_{\mathrm{L}} = 0.5$ and $p_{\mathrm{L}} = 0$, respectively. Similarly, when both inputs are 1, the two skyrmions are either at the LS or SL

state, which leads to a resistivity of $\langle\Delta\rho_{\mathrm{xy2}}\rangle \approx 0.008$ μΩ cm corresponding to an output of 1. When the input for one skyrmion is 0 and the other is 1, one skyrmion would be stabilized at the S state while the other has an increased probability at the L state, leading to a resistivity of $\langle\Delta\rho_{\mathrm{xy2}}\rangle \approx 0.008$ μΩ cm which corresponds to an output of 1, as well. When both inputs are 0, both skyrmions are at the S state, giving a low resistivity of $\langle\Delta\rho_{\mathrm{xy2}}\rangle \approx 0$ corresponding to the output of 0. Therefore, the logical operations can be achieved by local dynamics of two neighbouring skyrmions with an anti-correlated coupling. In previous studies, skyrmionic logics have been proposed on the basis of dynamic motion of skyrmions[18,19]. Implementing the skyrmion motion-based devices experimentally, however, encounters difficulties in the precise control over skyrmion motion, in geometric and operational complexities. Comparatively, the logic devices based on local dynamics of skyrmions proposed here are much easier to operate and more spatially compact. In addition to skyrmionic logics, two neighbouring skyrmions can serve as an element of a ternary numeral system with three discrete SS, LS/SL and LL states.

Two neighbouring skyrmions form the simplest skyrmion network. A more complex skyrmion network has further potential in computing. Skyrmion networks in naturally grown magnetic films have great potential in reservoir computing applications (Supplementary Fig. 13c). The reservoir computing paradigm is inspired by the human brain and is a type of recursive neural network which exhibits capabilities in recognition and prediction of spatio-temporal events. Reservoir computing does not require any knowledge of the node weights for training purposes, only the output weights, and can thus utilize the naturally existing skyrmion network in magnetic films. In skyrmion network-based reservoir computing, the applied fields or currents in particular regions serve as the inputs. Local magnetic fields can be in principle applied by multiple "monopole" writing elements and the local current can be applied through multiple nanocontacts. The skyrmion network with mutual interactions is the reservoir whose weighting function can be unknown, and the matrix of the node weights is generally fixed for reservoir computing. In a skyrmion device, a fixed matrix is generated under a given global field and current. When the field or current is varied, the matrix changes and a new reservoir computer is formed, thereby allowing the skyrmion network to have higher tunability and functionality compared to memristor and atomic-switch networks[20,21]. Electronic signals from selected regions can then be converted into output neurons.

In addition to naturally existing skyrmion networks, skyrmion lattices with different structures can also be implemented via artificial pinning centres. These may exhibit more interesting dynamics for potential in computing applications such as Ising computing. Overall, local dynamics of skyrmions may provide effective functionalities for versatile computing. Implementing these devices experimentally with demonstrations of skyrmion manipulations by using the local field and current remains to be further explored.

IX. Fluctuation rate in the local dynamics of a skyrmion

The fluctuation rate in the local dynamics of a skyrmion increases with increasing temperature, reducing energy barrier and the distance Δq between two weaker pinning sites. These dependencies can be theoretically understood through a forward flux sampling method which has been demonstrated to be effective in exploring thermal stability of skyrmions[22] and thermally activated magnetization reversals[23,24]. Although the energy profile can be described by two local minima at the two weaker pinning sites and an energy barrier in between, the recrossings will necessarily occur as the skyrmion approaches one state from the other, even in the vicinity of the barrier. The path sampling method relies on a series of interfaces in configuration space, $\{\lambda_{\mathrm{L}}, \lambda_0, \lambda_1, \cdots, \lambda_{\mathrm{n}-1}, \lambda_{\mathrm{n}} = \lambda_{\mathrm{S}}\}$, between the L and S states. These interfaces are defined as isosurfaces of a monotonically varying order parameter ζ. This order parameter can be the skyrmion size in the study of thermal stability of skyrmions[22] or the perpendicular magnetization m_{z} in the study of thermally activated magnetization reversals[23,24]. In the skyrmion size fluctuation dynamics, the order parameter can also be defined by the perpendicular magnetization. The fluctuation rate of the skyrmion is then expressed as $f = \Phi_{\mathrm{L},0} \prod_{i=0}^{\mathrm{n}-1} P(\lambda_{i+1}|\lambda_i)$ where $\Phi_{\mathrm{L},0}$ represents the flux of trajectories from L to the first interface λ_0 and $P(\lambda_{i+1}|\lambda_i)$ represents the probability that a trajectory starting at λ_i reaches λ_{i+1} before returning to the basin L state. $\Phi_{\mathrm{L},0}$ can be estimated through $\Phi_{\mathrm{L},0} = N_0/\Delta t_{\mathrm{sim}}$ where N_0 is the number of configurations at λ_0 obtained starting from the basin L in the total simulation time of Δt_{sim}. In general, $P(\lambda_{i+1}|\lambda_i)$ increases with λ_i. With increasing Δq, more interfaces are needed to cross from the basin L to S, reducing $\prod_{i=0}^{\mathrm{n}-1} P(\lambda_{i+1}|\lambda_i)$ and thus the fluctuation rate. On the other hand, $\Phi_{\mathrm{L},0}$ is expected to be larger at an elevated temperature and with a reduced barrier height, which will thereby increase the fluctuation rate. A deeper understanding of how the fluctuation rate scales with temperature, barrier height, and Δq remains to be further explored which is beyond the scope of this paper.

X. Micromagnetic simulations of local dynamics of skyrmions

We perform micromagnetic simulations using finite-difference solver MUMAX3 based on the graphic processing unit[25]. Supplementary Figure 14 shows simulation results of two separate skyrmions with each one fluctuating in time between the S and L states. Supplementary Figure 14a, e, show magnetic configurations of the S state (the left panel) and L state (the right panel) of the skyrmions. The transition between the S and L states of the skyrmions is dominated by the thermal motion of the part of the skyrmions in the blue box region. Supplementary Figure 14b, f, display the magnetization profiles along the red arrow for the

magnetic configurations shown in Supplementary Fig. 14a, e. The mobile part of both skyrmions can fluctuate in time between two sites, leading to the fluctuation of the perpendicular magnetization m_z in the blue box region as shown in Supplementary Fig. 14c, g.

For the skyrmion shown in Supplementary Fig. 14a-c, the mobile part of the skyrmion fluctuating in time between two sites separated by approximately 50 nm and the average residence time τ is as low as 30 ns. On the other hand, for the skyrmion presented in Supplementary Fig. 14e-g, this time is about 540 ns while the distance between the two weaker pinning sites is larger and about 60 nm. We summarize the average residence time τ of both skyrmions as functions of $\Delta K_u/K_u$, the random PMA variation, and temperature T, in Supplementary Fig. 14d, h. $\Delta K_u/K_u$ reflects the energy barrier height and thus a lower τ is observed at a lower $\Delta K_u/K_u$. The simulation results indicate that a fluctuation rate beyond the MHz range may be experimentally achieved by more elaborate control of the energy landscape, which remains to be further studied.

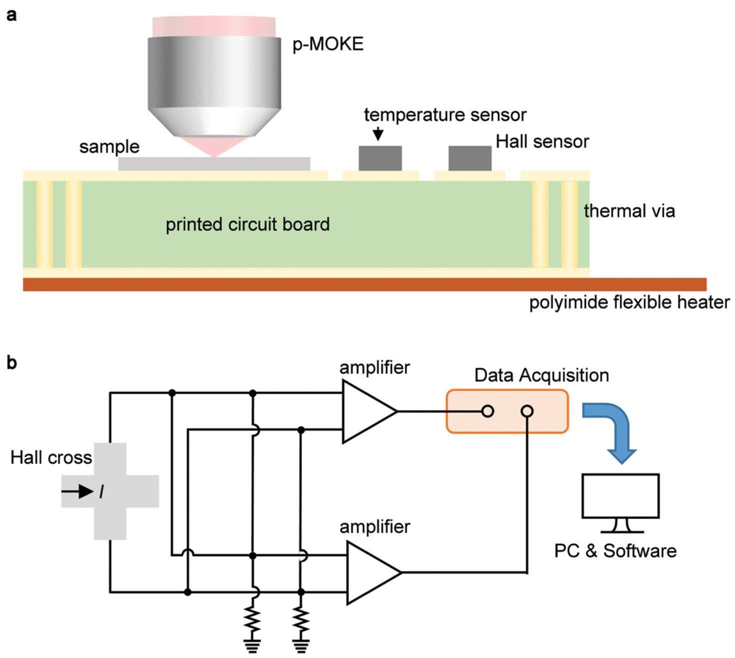

Supplementary Figure 1: **Experimental setup. a**, Schematic of the experimental setup. **b**, Circuit diagram of electronic measurements.

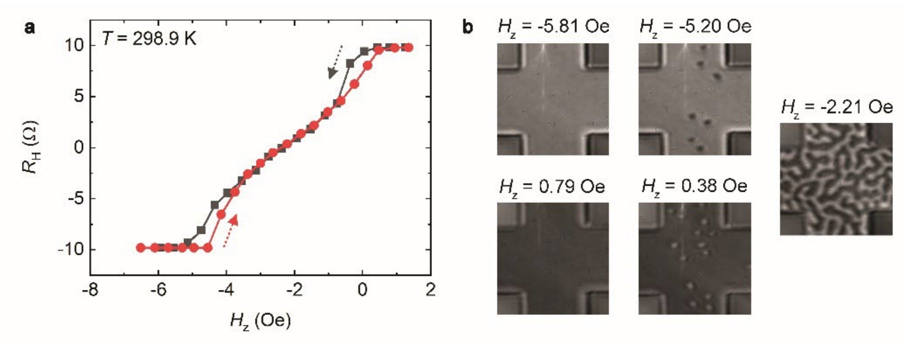

Supplementary Figure 2: **Magnetic properties of the magnetic film. a**, Hall resistance $\boldsymbol{R}_{\mathbf{H}}$ versus the perpendicular magnetic field H_z. Arrows represent sweeping directions of the magnetic field. **b**, P-MOKE images of the magnetic film at different fields. The bright and dark contrasts, respectively, correspond to downward and upward out-of-plane magnetizations. The Hall cross in has dimensions $20 \times 20\ \mu m^2$.

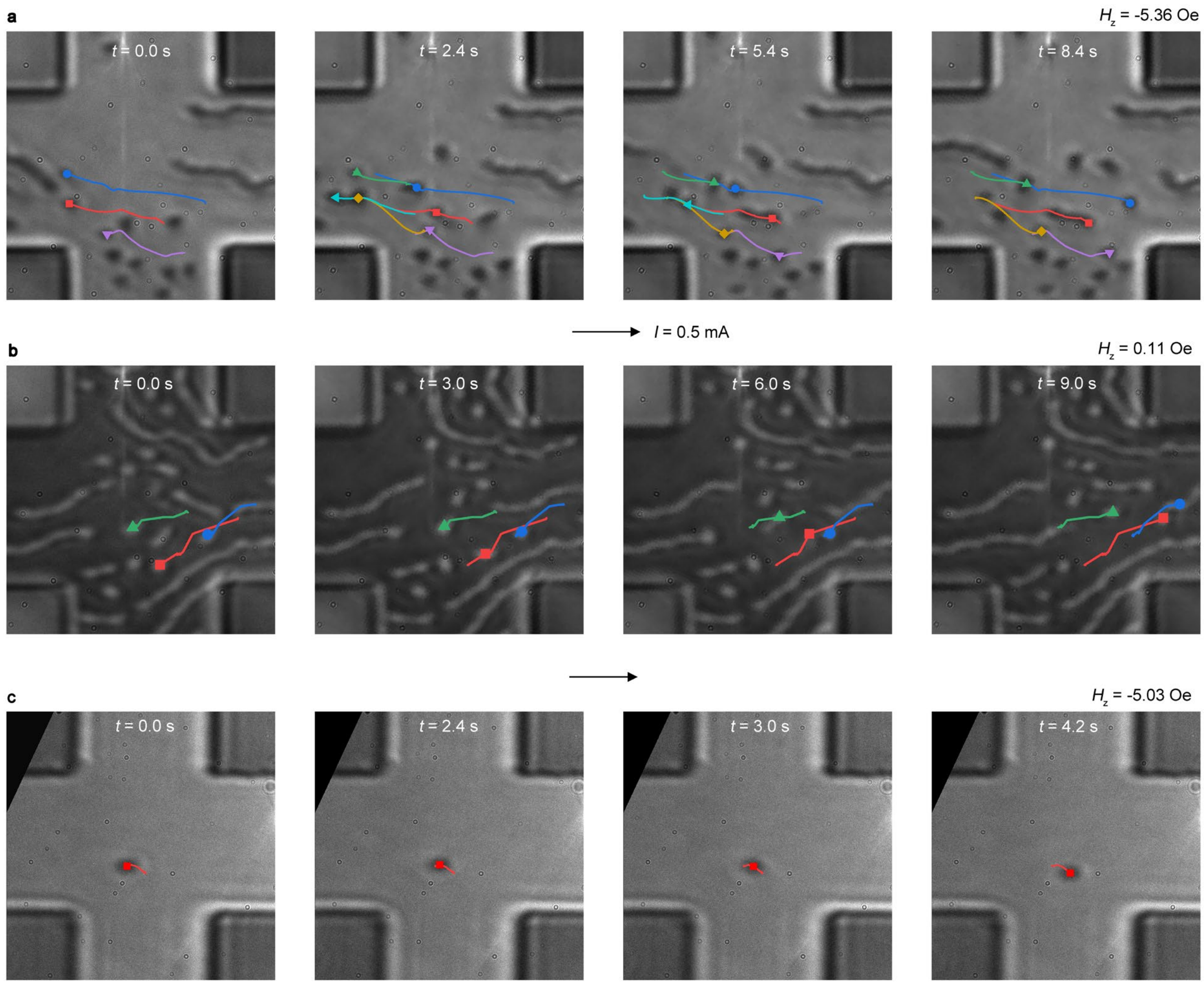

Supplementary Figure 3: **Skyrmion Hall effect. a**, **b**, the dynamic motion of skyrmions with opposite polarities in the presence of a constant current of 0.5 mA (the black arrow). Lines are trajectories of skyrmions under this current. Symbols are positions of skyrmions at the corresponding time of each image. The bright and dark contrasts, respectively, correspond to downward and upward out-of-plane magnetizations. The Hall cross has dimensions 20×20 μm^2. **c**, the dynamic motion of the skyrmion shown in Fig. 1b-d in the main text under a driving current (the black arrow) by which the skyrmion escapes from the local pinning centre and experiences the skyrmion Hall effect.

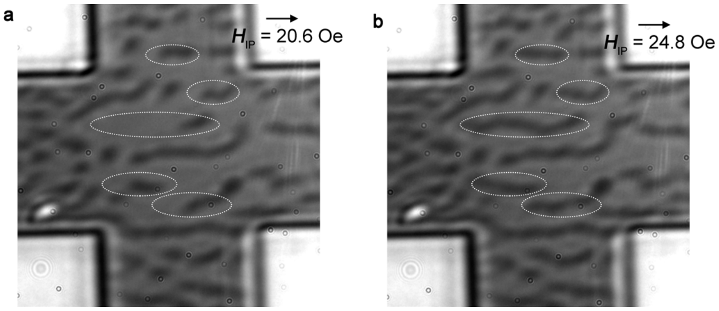

Supplementary Figure 4: **Determination of chirality and vorticity of magnetic domain walls.** Magnetic configurations recorded when applying an in-plane magnetic field $H_{\mathrm{IP}} = 20.6$ Oe (**a**) and $H_{\mathrm{IP}} = 24.8$ Oe (**b**) along the black arrow when a perpendicular magnetic field $\mathrm{H_z} = -6.43$ Oe is applied.

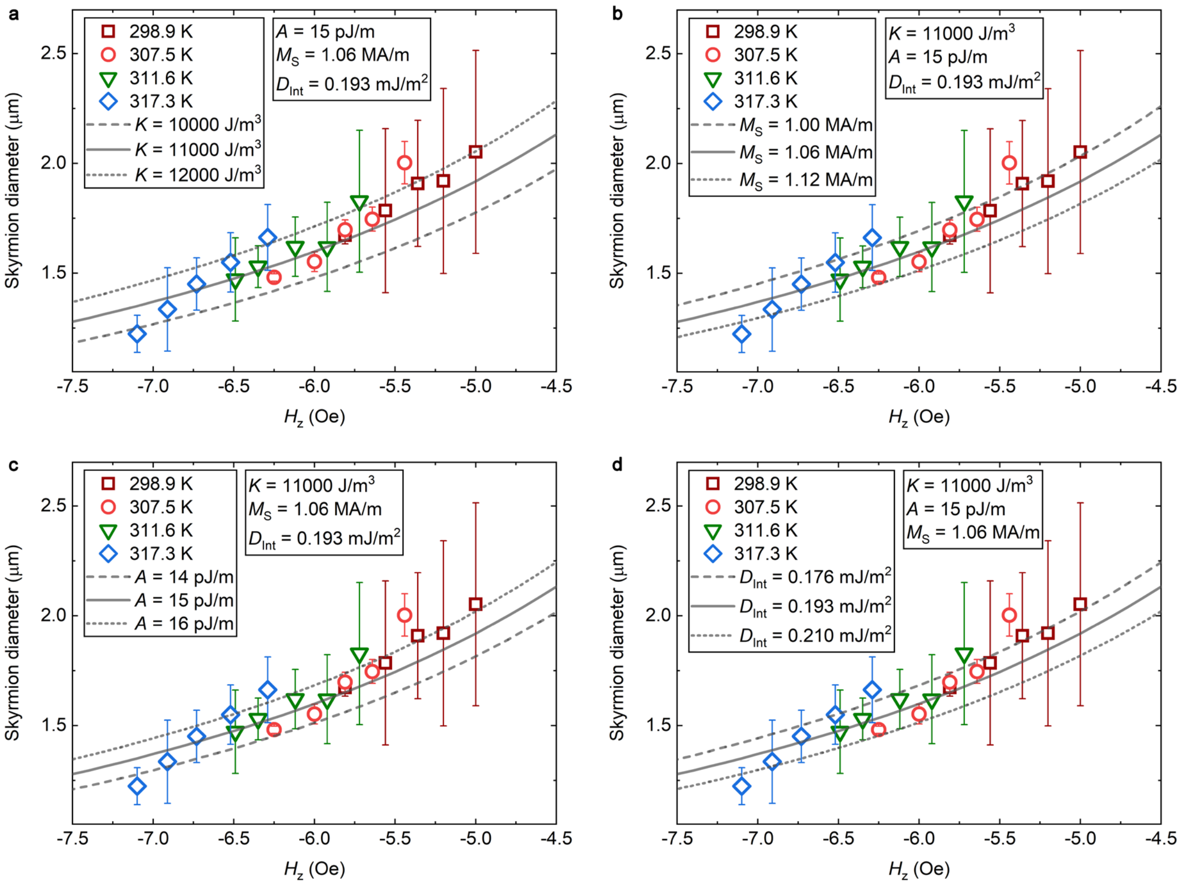

Supplementary Figure 5: **Skyrmion sizes.** The skyrmion diameter derived from p-MOKE images recorded at different perpendicular fields H_z and temperatures. Lines are theoretical calculations of the skyrmion diameter with adopting different parameters of the effective perpendicular magnetic anisotropy K, saturation magnetization M_S, exchange stiffness A, and the interfacial DMI D_{Int}. In calculations of the skyrmion diameter in **a-d**, K, M_S, A, and D_{Int} parameters are respectively varied while others are fixed.

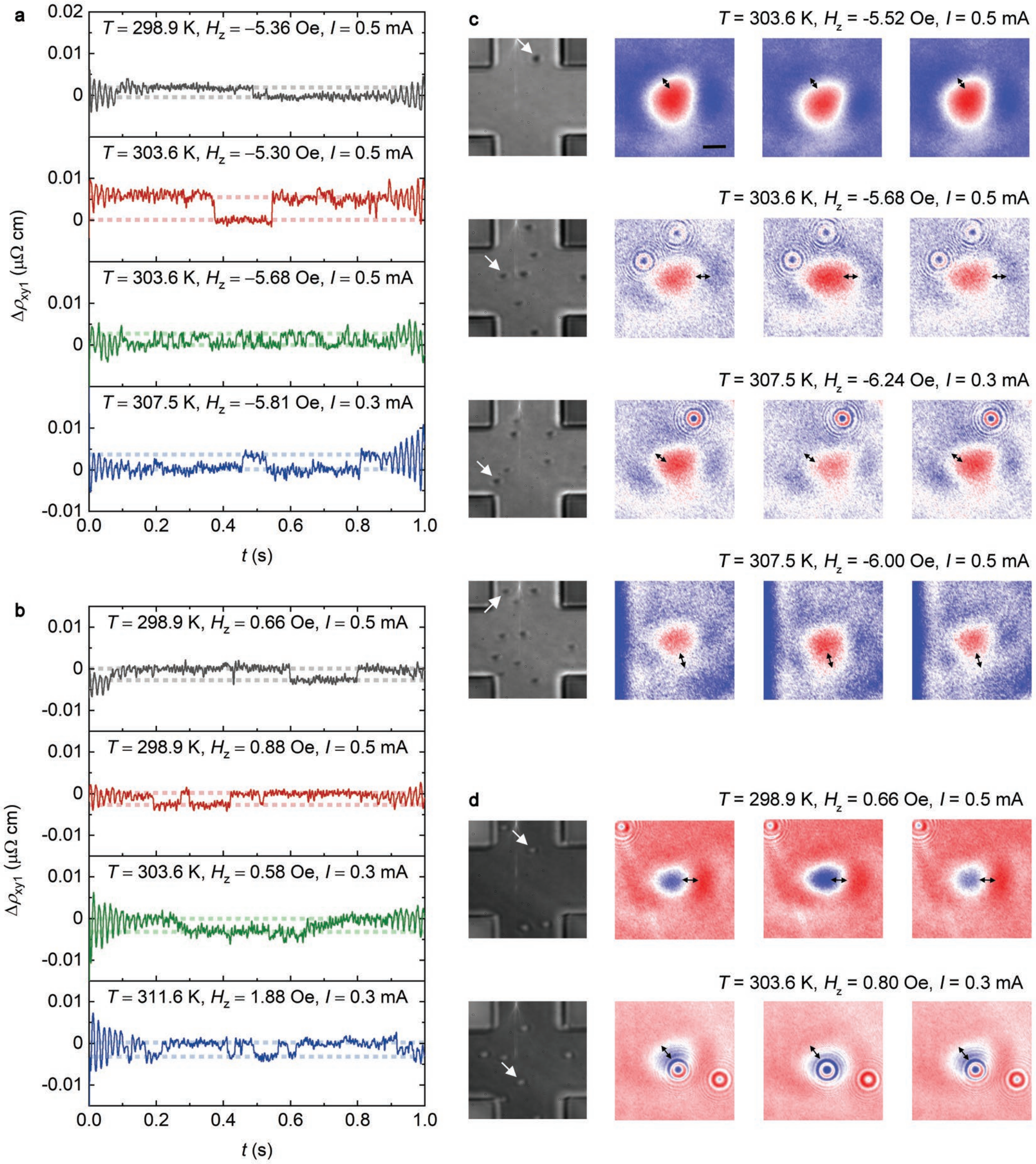

Supplementary Figure 6: **Local dynamics of skyrmions.** **a**, Fluctuations of the relative Hall resistivity $\Delta\rho_{\mathrm{xy1}}$ observed in multiple measurements for skyrmions with the polarity of 1. **b**, Fluctuations of the relative Hall resistivity $\Delta\rho_{\mathrm{xy1}}$ observed in multiple measurements for skyrmions with the polarity of -1. **c**, P-MOKE images for skyrmions with the polarity of 1. **d**, P-MOKE images for skyrmions with the polarity of -1. The right three images in each row are enlarged images of the skyrmion marked by the white arrow in the most left p-MOKE image. The black double-headed arrows represent the motion of the mobile part of the skyrmion. The bright (blue) and dark (red) contrasts in **c**, **d**, respectively, correspond to downward and upward out-of-plane magnetizations. The length of the scale bar in **c** represents 1 μm.

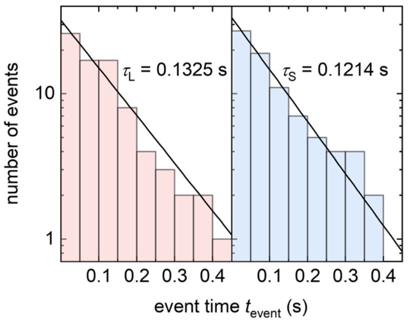

Supplementary Figure 7: **Histogram of the switching event time.** Histogram of the residence time for the L and S states collected by monitoring signals in 21 s. The results are analysed at $H_z = -5.75$ Oe, $I = -0.2$ mA and $T = 307.1$ K. Lines are exponential fittings from which the average residence times τ_L and τ_S are derived.

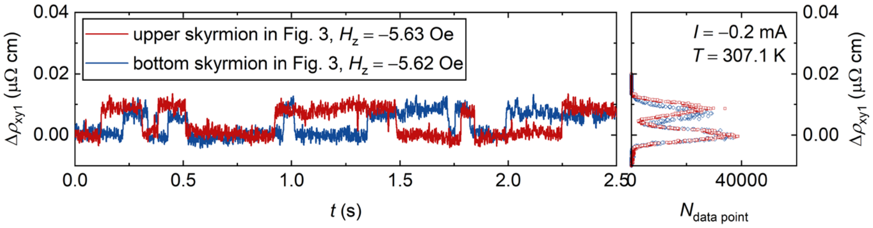

Supplementary Figure 8: **Local dynamics of each skyrmion of two neighbouring skyrmions.** Relative Hall resistivity $\Delta\rho_{\mathrm{xy1}}$ variations in time for each skyrmion of the two neighbouring skyrmions shown in Fig. 3 in the main text. The right panel shows the statistics of data point distributions for the two skyrmions.

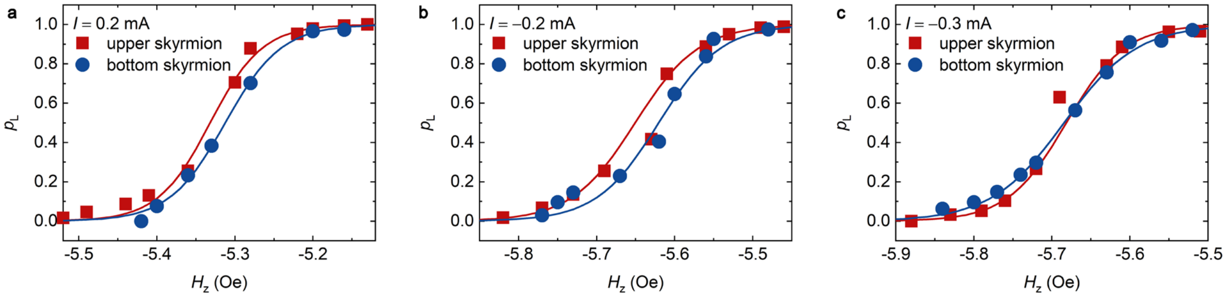

Supplementary Figure 9: **Field and current control of the switching.** Field H_z dependences of the switching probability p_L for each skyrmion of the two neighbouring skyrmions shown in Fig. 3 in the main text. Results in **a-c** are measured at a fixed current of 0.2, -0.2 and -0.3 mA, respectively. The results show the similar field dependence of the switching probability at different currents for the two skyrmions.

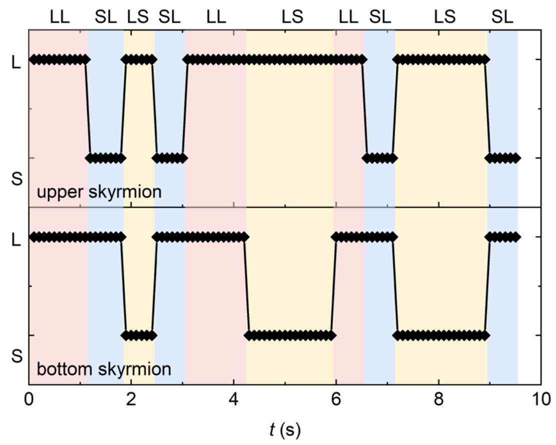

Supplementary Figure 10: **Fluctuations of two neighbouring skyrmions in time.** Fluctuations of the two neighbouring skyrmions shown in Fig. 3a-c in time between the LL, LS, SL and SS states, extracted from p-MOKE measurements (Supplementary movie 5). Note that the fluctuation rate shown in this figure is limited by the time resolution in p-MOKE measurements. The two skyrmions fluctuate in the most time between the LS and SL states, inferring that the two skyrmions exhibit an anti-correlated coupling in their fluctuation dynamics, consistent with the electronic measurements (Fig. 3d in the main text). The results are measured at 307.1 K, $H_{\mathrm{z}} = -5.60$ Oe and $I = -0.2$ mA. The switching probability $p_{\mathrm{LS/SL}} \approx 68.8\%$ is derived from this figure, which is also consistent with that derived from electronic measurements (Fig. 3f in the main text).

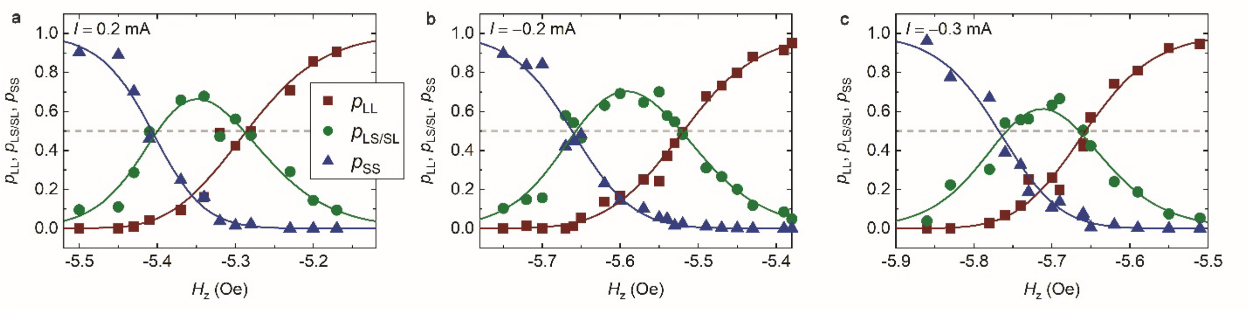

Supplementary Figure 11: **Field and current control of the switching for two neighbouring skyrmions.** Field H_z dependences of the switching probabilities p_{LL} (square), $p_{LS/SL}$ (circle) and p_{SS} (up triangle) at the LL, LS/SL and SS state, respectively, measured at different currents. For each set of measurement results at a fixed current, the $p_{LS/SL}$ is above 0.5 at the middle field. Considering the two skyrmions, independently, have the same field and current dependences of the switching (Supplementary Fig. 9), the results suggest that the two skyrmions exhibit an anti-correlated coupling as discussed in the main text.

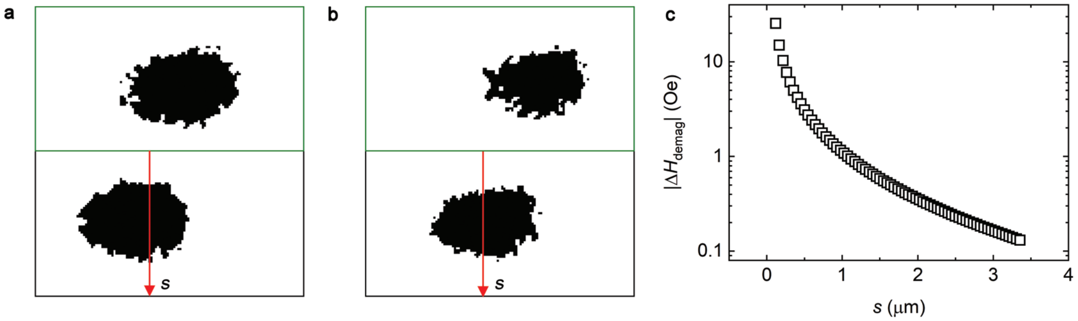

Supplementary Figure 12: **Demagnetization-field variation owing to the fluctuation in skyrmion size.** **a**, **b** Maps of the perpendicular magnetization m_{z} for the LL-state (**a**) and for the SS-state (**b**) skyrmion, derived from P-MOKE images shown in Fig. 3a, c, respectively. $m_{\mathrm{z}} = 1$ in the black region and $m_{\mathrm{z}} = -1$ in the white region. **c**, The demagnetization-field variation $|\Delta H_{\mathrm{demag}}|$ at different positions along the red arrow in **a**, **b** owing to changes in magnetic configurations in green square regions.

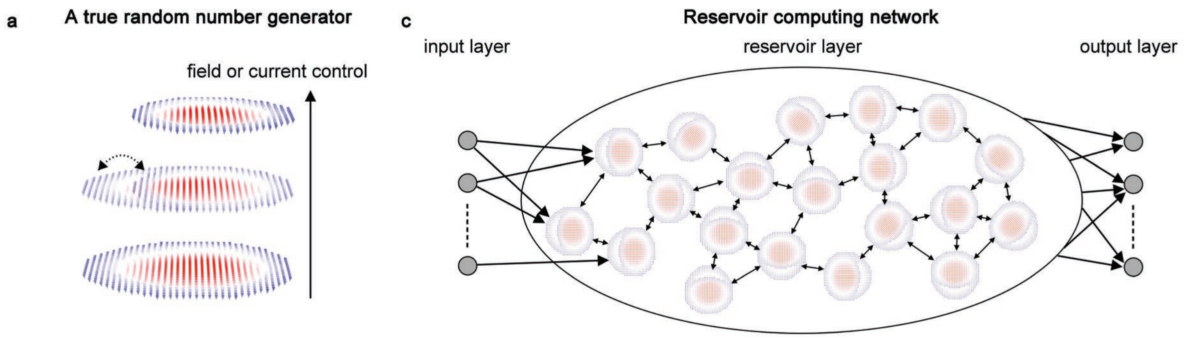

b

AND

input		output		
current	code	magnetic configuration	<$\Delta\rho_{xy1}$> (μΩ cm)	code
⟵<br>⟵	1<br>1	LL	0.016	1
⟵<br>←	1<br>0	LS	0.008	0
←<br>⟵	0<br>1	SL	0.008	0
←<br>←	0<br>0	LS/SL	0.008	0

OR

input		output		
current	code	magnetic configuration	<$\Delta\rho_{xy1}$> (μΩ cm)	code
⟵<br>⟵	1<br>1	LS/SL	0.008	1
⟵<br>←	1<br>0	LS	0.008	1
←<br>⟵	0<br>1	SL	0.008	1
←<br>←	0<br>0	SS	0.000	0

Supplementary Figure 13: **Potential of local dynamics of skyrmions for applications in computing. a**, A true random number generator based on local dynamics of a single magnetic skyrmion. **b**, Logical AND and OR operations based on two neighbouring skyrmions with an anti-correlated coupling. **c**, The reservoir computing based on a naturally existing skyrmion network in magnetic films. See Supplementary Note VIII for more discussions.

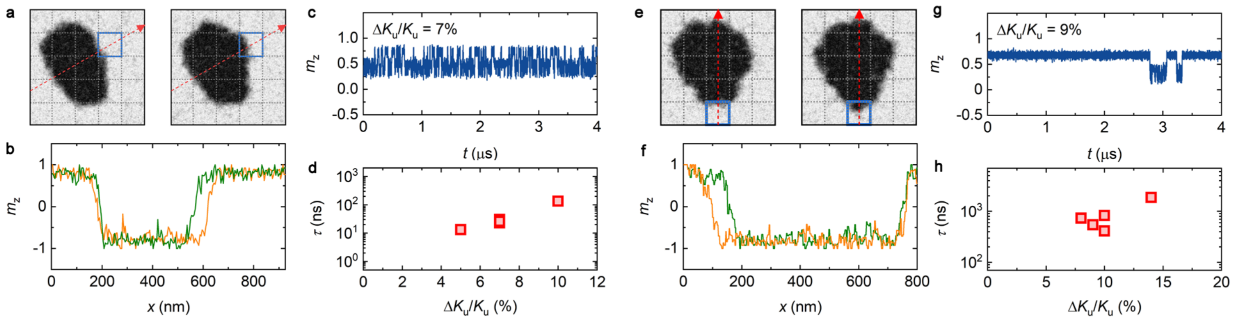

Supplementary Figure 14: **Local dynamics of skyrmions in micromagnetic simulations.** Figures in **a-d** and **e-h** are results for two skyrmions respectively. **a**, **e**, Maps of the perpendicular magnetization m_z for the skyrmions with the S (left panel) and L states (right panel). $m_z = -1$ in the black region and $m_z = 1$ in the white region. **b**, **f**, m_z variations along the red arrow in **a** and **e**, respectively. Both magnetization profiles of the S-state and L-state skyrmions are shown. **c**, **g**, Fluctuations of the average m_z in the blue square region in **a** and **e**, respectively. **d**, **h**, the average fluctuation rate of the skyrmions as a function of $\Delta K_u/K_u$. $T = 300$ K is adopted in simulations.

Supplementary movie 1

P-MOKE movie of the creep motion of magnetic domains and skyrmions

This movie is recorded by the p-MOKE microscope on the magnetic film grown with the DC power $P_{\mathrm{Ta}} = 4$ Watt for the deposition of the Ta layer. This movie is acquired at a perpendicular magnetic field $H_{\mathrm{z}} = -5.05$ Oe, at a constant current $I = 0.5$ mA and at the temperature $T = 307.5$ K. The temporal resolution of the movie is 0.61 s.

Supplementary movie 2

P-MOKE movie of the steady flow of magnetic domains and skyrmions

This movie is recorded by the p-MOKE microscope on the magnetic film grown with the DC power $P_{\mathrm{Ta}} = 5$ Watt for the deposition of the Ta layer. This movie is acquired at a perpendicular magnetic field $H_{\mathrm{z}} = -2.65$ Oe and at the temperature $T = 305.8$ K. In the first two seconds, no current is applied. Thermal fluctuations of magnetic domains and skyrmions are observed. Afterwards a constant current $I = 0.5$ mA is applied, under which the magnetic domains and skyrmions flow steadily. The temporal resolution of the movie is 0.61 s.

Supplementary movie 3

P-MOKE movie of the thermal motion of a skyrmion

This movie is recorded by the p-MOKE microscope on the magnetic film grown with the DC power $P_{\mathrm{Ta}} = 5$ Watt for the deposition of the Ta layer. This movie is acquired at a perpendicular magnetic field $H_{\mathrm{z}} = -3.52$ Oe and at the temperature $T = 312.7$ K. No current is applied during the measurement. Thermal motion of a skyrmion is observed.

Supplementary movie 4

P-MOKE movie of the local dynamics of a single skyrmion

This movie is recorded by the p-MOKE microscope on the magnetic film grown with the DC power $P_{\mathrm{Ta}} = 4$ Watt for the deposition of the Ta layer. This movie is acquired at a perpendicular magnetic field $H_{\mathrm{z}} = -5.76$ Oe, at a constant current $I = -0.2$ mA and at the temperature $T = 307.1$ K. The temporal resolution of the movie is 0.61 s. The fluctuation of the skyrmion in time between the S and L states is observed.

Supplementary movie 5

P-MOKE movie of the local dynamics of two neighboring skyrmion

This movie is recorded by the p-MOKE microscope on the magnetic film grown with the DC power $P_{\mathrm{Ta}} = 4$ Watt for the deposition of the Ta layer. This movie is acquired at a perpendicular magnetic field $H_{\mathrm{z}} = -5.65$ Oe, at a constant current $I = -0.2$ mA and at the temperature $T = 307.1$ K. The temporal resolution of the movie is 0.61 s. The fluctuation of both skyrmions in time between the S and L states is observed.

Supplementary References

1 Fan, Y. *et al.* Exchange bias of the interface spin system at the Fe/MgO interface. *Nat. Nanotechnol.* **8**, 438-444 (2013).

2 Xu, M. *et al.* Voltage-Controlled Antiferromagnetism in Magnetic Tunnel Junctions. *Phys. Rev. Lett.* **124**, 187701 (2020).

3 Jiang, W. *et al.* Direct observation of the skyrmion Hall effect. *Nat. Phys.* **13**, 162-169 (2017).

4 Litzius, K. *et al.* Skyrmion Hall effect revealed by direct time-resolved X-ray microscopy. *Nat. Phys.* **13**, 170-175 (2017).

5 Thiele, A. Steady-state motion of magnetic domains. *Phys. Rev. Lett.* **30**, 230 (1973).

6 Wang, K., Qian, L., Ying, S.-C., Xiao, G. & Wu, X. Controlled modification of skyrmion information in a three-terminal racetrack memory. *Nanoscale* **11**, 6952-6961 (2019).

7 Lemesh, I., Büttner, F. & Beach, G. S. Accurate model of the stripe domain phase of perpendicularly magnetized multilayers. *Phys. Rev. B* **95**, 174423 (2017).

8 Wang, K. *et al.* Spin torque effect on topological defects and transitions of magnetic domain phases in Ta/CoFeB/MgO. *Phys. Rev. B* **99**, 184410 (2019).

9 Zhang, S., Zhang, J., Wen, Y., Chudnovsky, E. M. & Zhang, X. Determination of chirality and density control of Néel-type skyrmions with in-plane magnetic field. *Commun. Phys.* **1**, 1-7 (2018).

10 Jiang, W. *et al.* Blowing magnetic skyrmion bubbles. *Science* **349**, 283-286 (2015).

11 Wang, X., Yuan, H. & Wang, X. A theory on skyrmion size. *Commun. Phys.* **1**, 1-7 (2018).

12 Qian, L., Wang, K., Zheng, Y. & Xiao, G. Spin Hall effect in the α and β phases of T a x W 1− x alloys. *Phys. Rev. B* **102**, 094438 (2020).

13 Hayakawa, K. *et al.* Nanosecond Random Telegraph Noise in In-Plane Magnetic Tunnel Junctions. *Phys. Rev. Lett.* **126**, 117202 (2021).

14 Borders, W. A. *et al.* Integer factorization using stochastic magnetic tunnel junctions. *Nature* **573**, 390-393 (2019).

15 Zhang, S. *et al.* Direct writing of room temperature and zero field skyrmion lattices by a scanning local magnetic field. *Appl. Phys. Lett.* **112**, 132405 (2018).

16 Casiraghi, A. *et al.* Individual skyrmion manipulation by local magnetic field gradients. *Commun. Phys.* **2**, 1-9 (2019).

17 Woo, S. *et al.* Observation of room-temperature magnetic skyrmions and their current-driven dynamics in ultrathin metallic ferromagnets. *Nat. Mater.* **15**, 501-506 (2016).
18 Zhang, S., Baker, A. A., Komineas, S. & Hesjedal, T. Topological computation based on direct magnetic logic communication. *Sci. Rep.* **5**, 1-11 (2015).
19 Zhang, X., Ezawa, M. & Zhou, Y. Magnetic skyrmion logic gates: conversion, duplication and merging of skyrmions. *Sci. Rep.* **5**, 1-8 (2015).
20 Sillin, H. O. *et al.* A theoretical and experimental study of neuromorphic atomic switch networks for reservoir computing. *Nanotechnology* **24**, 384004 (2013).
21 Stieg, A. Z. *et al.* Emergent criticality in complex turing B - type atomic switch networks. *Adv. Mater.* **24**, 286-293 (2012).
22 Vansteenkiste, A. *et al.* The design and verification of MuMax3. *AIP Adv.* **4**, 107133 (2014).
