# Supplementary material for: Single skyrmion true random number generator using local dynamics and interaction between skyrmions
Source: Nat Commun. 2022 Feb 7;13:722. doi: 10.1038/s41467-022-28334-4 (PMC8821635; doi:10.1038/s41467-022-28334-4)
Supplement: Supplementary file 7 — Description of Additional Supplementary Files [file 41467_2022_28334_MOESM7_ESM.pdf]

Title: Supplementary movie 1
Description: P-MOKE movie of the creep motion of magnetic domains and

skyrmions This movie is recorded by the p-MOKE microscope on the magnetic film grown with the DC power PPTa = 4 Watt for the deposition of the Ta layer. This movie is acquired at a perpendicular magnetic field HHz = −5.05 Oe, at a constant current II = 0.5 mA and at the temperature TT = 307.5 K. The temporal resolution of the movie is 0.61 s.

Title: Supplementary movie 2
Description: P-MOKE movie of the steady flow of magnetic domains and

skyrmions This movie is recorded by the p-MOKE microscope on the magnetic film grown with the DC power PPTa = 5 Watt for the deposition of the Ta layer. This movie is acquired at a perpendicular magnetic field HHz = −2.65 Oe and at the temperature TT = 305.8 K. In the first two seconds, no current is applied. Thermal fluctuations of magnetic domains and skyrmions are observed. Afterwards a constant current II = 0.5 mA is applied, under which the magnetic domains and skyrmions flow steadily. The temporal resolution of the movie is 0.61 s.

Title: Supplementary movie 3
Description: P-MOKE movie of the thermal motion of a

skyrmion This movie is recorded by the p-MOKE microscope on the magnetic film grown with the DC power PPTa = 5 Watt for the deposition of the Ta layer. This movie is acquired at a perpendicular magnetic field HHz = −3.52 Oe and at the temperature TT = 312.7 K. No current is applied during the measurement. Thermal motion of a skyrmion is observed.

Title: Supplementary movie 4
Description: P-MOKE movie of the local dynamics of a single skyrmion

This movie is recorded by the p-MOKE microscope on the magnetic film grown with the DC power PPTa = 4 Watt for the deposition of the Ta layer. This movie is acquired at a perpendicular magnetic field HHz = −5.76 Oe, at a constant current II = −0.2 mA and at the temperature TT = 307.1 K. The temporal resolution of the movie is 0.61 s. The fluctuation of the skyrmion in time between the S and L states is observed. 26

Title: Supplementary movie 5
Description: P-MOKE movie of the local dynamics of two neighboring skyrmion

This movie is recorded by the p-MOKE microscope on the magnetic film grown with the DC power PPTa = 4 Watt for the deposition of the Ta layer. This movie is acquired at a perpendicular magnetic field HHz = −5.65 Oe, at a constant current II = −0.2 mA and at the temperature TT = 307.1 K. The temporal resolution of the movie is 0.61 s. The fluctuation of both skyrmions in time between the S and L states is observed.
